# The role of COP1 in repression of photoperiodic flowering

**DOI:** 10.12688/f1000research.7346.1

**Published:** 2016-02-16

**Authors:** Dongqing Xu, Danmeng Zhu, Xing Wang Deng

**Affiliations:** 1 State Key Laboratory of Protein and Plant Gene Research, Peking-Tsinghua Center for Life Sciences, School of Advanced Agriculture Sciences and School of Life Sciences, Peking University, Beijing, China

**Keywords:** COP1, photoperiodic flowering, CONSTITUTIVE PHOTOMORPHOGENIC 1, flowering time

## Abstract

Plants use the circadian clock as a timekeeping mechanism to regulate photoperiodic flowering in response to the seasonal changes. CONSTITUTIVELY PHOTOMORPHOGENIC 1 (COP1), initially identified as a central repressor of seedling photomorphogenesis, was recently shown to be involved in the regulation of light input to the circadian clock, modulating the circadian rhythm and flowering. COP1 encodes a RING-finger E3 ubiquitin ligase and works in concert with SUPPRESSOR of *phyA-105* (SPA) proteins to repress photoperiodic flowering by regulating proteasome-mediated degradation of CONSTANS (CO), a central regulator of photoperiodic flowering. In addition, COP1 and EARLY FLOWERING 3 (ELF3) indirectly modulate *CO* expression via the degradation of GIGANTEA (GI). Here, we summarize the current understanding of the molecular mechanisms underlying COP1’s role in controlling of photoperiodic flowering.

## Introduction

In plants, the phase transition from vegetative to reproductive development is controlled by multiple environmental cues, including photoperiod, light quality, and temperature ^1^. According to their flowering response to the photoperiod change, plants could be classified as long-day (LD) plants, short-day (SD) plants, and day-neutral plants, respectively ^2^. At present, most advances regarding the flowering-time control were obtained in the model facultative LD plant *Arabidopsis* and the model SD plant rice. A central regulator of LD-induced flowering is the B-box zinc finger transcription factor CONSTANS (CO), which positively regulates flowering time by upregulating the expression of “florigen” *FLOWERING LOCUS T* ( *FT*) in *Arabidopsis* ^3^. The control of CO abundance by circadian clock and light plays a crucial role in regulating flowering.

CONSTITUTIVELY PHOTOMORPHOGENIC 1 (COP1) was initially identified as a key repressor of photomorphogenesis over 20 years ago in *Arabidopsis* ^4, 5^. The subsequent characterization of COP1 revealed its function in multiple light-mediated developmental processes in *Arabidopsis* and other higher plants, including circadian rhythm and flowering ^6, 7^. The ortholog of *Arabidopsis* COP1 was also found to play vital roles in regulating a variety of developmental processes in animals. COP1 encodes a RING-finger E3 ubiquitin ligase. In *Arabidopsis*, COP1 functions together with SUPPRESSOR of *phyA-105* (SPA) proteins to target the photomorphogenesis-promoting factors for degradation via the 26S proteasome system, such as ELONGATED HYPOCOTYL 5 (HY5), LONG AFTER FAR-RED LIGHT 1 (LAF1), and LONG HYPOCOTYL IN FAR-RED 1 (HFR1) ^8– 11^.

## The relationship of photoreceptors and COP1 in flowering

In *Arabidopsis*, far-red and red light is perceived by phytochromes (phyA-phyE) ^12, 13^; blue light is sensed by cryptochromes (CRY1 and CRY2) and several new photoperiodic and/or circadian photoreceptors: ZEITLUPE (ZTL), FLAVIN-BINDING, KELCH REPEAT, F-BOX 1 (FKF1), and LOV, KELCH PROTEIN 2 (LKP2) ^14^. It was reported that phyA and CRYs are two classes of principal photoperiodic photoreceptors that promote flowering. Mutations in these genes reduce the accumulation of CO protein and delay flowering ^15, 16^. During photomorphogenesis, CRYs suppress the activity of the multifunctional E3 ubiquitin ligase COP1 by dissociating the formation of COP1-SPA complex(es), thereby repressing its E3 ubiquitin ligase activity to regulate gene expression in response to blue light ^17– 19^. In flowering transition, blue light-dependent CRY2-SPA1 interaction stimulates CRY2-COP1 association to suppress the COP1-dependent proteolysis of CO ^19^. However, how phyA mediates light regulation of protein degradation to modulate developmental timing in flowering is unclear at present. In contrast to *cry2*, the early-flowering phenotype of *phyB* in SD is possibly resulting from a COP1-independent mechanism ^15, 16, 20^. Paradoxically, plants overexpressing phyB also show early flowering, in which the Pfr form of phyB inhibits COP1-SPA activity to stabilize CO and subsequently induce *FT* expression by phyB-SPA1 direct interaction ^21^.

## COP1 direct targets in modulation of flowering

CO acts as a central regulator of photoperiodic flowering, and its abundance directly correlates with the timing of flowering. CO is precisely regulated at both transcriptional and post-translational levels, and this is crucial for *Arabidopsis* to discriminate the photoperiod and response to light.

The expression of *CO* is regulated by circadian clock-associated components, including GIGANTEA (GI), the F-box protein FKF1, and CYCLING DOF FACTORS (CDFs), which regulate daily *CO* expression profiles ^22– 24^. EARLY FLOWERING 3 (ELF3) acts as a substrate adaptor to allow COP1-GI interaction, which leads to the degradation of GI by COP1 ^25^. FKF1 forms a complex with GI in a light-dependent manner, which contributes to control the *CO* transcript level by mediating the degradation of *CO* transcriptional repressors, CDFs ^22– 24^. Thus, degradation of GI by COP1 may result in the disassociation of FKF1-GI complex and then negatively regulate *CO* expression.

Post-translational regulation of CO is another aspect for controlling flowering in response to day length. *cop1* mutants display early-flowering phenotype under SD, which is largely related to the change of CO abundance. During the day, CO protein is stabilized, whereas at night CO protein is rapidly degraded through the 26S proteasome pathway mediated by COP1. COP1 directly interacts with the C-terminal of CO in phloem companion cells, where FT protein moves to induce flowering at the shoot apex ^26, 27^. In addition, the early-flowering phenotype of *spa1* is enhanced by the lesion in *SPA3* and *SPA4*. SPA proteins negatively modulate CO abundance so that *spa1 spa3 spa4* triple mutants exhibit strongly increased CO protein levels ^28^. A recent report further demonstrated that the COP1-SPA complex(es) directly interact with the phosphorylated form of CO protein to trigger its protein turnover ^29^.

In the early morning, TARGET OF EAT (TOE) proteins associate with the transcriptional activation domain of CO to inhibit its activity ^30^. FKE1 stabilizes the CO abundance through a direct interaction in the late afternoon of LD ^31^. At night, CO is degraded through the ubiquitin-mediated 26S proteasome system. Consistently, CO protein levels and its direct target *FT* peak in the afternoon under LD conditions ^32^. CO activates *FT* expression mainly through two modes of action: (1) CO directly binds to the CO-responsive element (CORE) in the promoter of *FT* to activate its expression ^33^. (2) CO physically interacts with two other *FT* activators NUCLEAR FACTOR-Y (NF-Y) and Myb transcription factor ASYMMETRIC LEAVES 1 (AS1), which directly bind to *FT* promoter, thus promoting their activation on *FT* ^34, 35^. COP1 triggers the protein turnover of CO, in turn disrupting the formation of CO-NF-Y and CO-AS1 complexes and eventually repressing the *FT* expression.

Besides light, temperature is another important environmental indicator to determine the appropriate time to flower. Recent work showed that COP1 could act as an integrator of light and cold temperature. *cop1* mutants exhibit reduced sensitivity to changes in ambient temperatures in an *FT*-dependent manner in *Arabidopsis*. At low ambient temperatures, COP1 is stabilized and subsequently promotes the degradation of GI, which directly activates *FT* expression to promote flowering ^36^.

## COP1-related factors in control of flowering

Similar to COP1, another repressor of photomorphogenesis, DE-ETIOLATE 1 (DET1), functions as a negative regulator of flowering, as *det1* mutants flower early in both LD and SD (extremely early in SD) ^37^. DET1 was shown to be part of the COP10, DE-ETIOLATE 1, DAMAGED DNA-BINDING PROTEIN 1 (CDD) complex, working as CUL4-based E3 ligase ^38^. Co-suppression mutants of *CUL4* also showed early-flowering phenotype under SD conditions. CUL4-DDB1 also associates with COP1-SPA complexes ^39^. Together, these studies indicate that a series of E3 ligase complexes may work in concert to repress flowering.

Recent studies revealed that, besides COP1, another RING-finger containing E3 ubiquitin ligase, HIGH EXPRESSION OF OSMOTICALLY RESPONSIVE GENES 1 (HOS1), is also involved in controlling the CO protein levels. In the morning of LD, phyB-mediated red light signaling activates HOS1 to degrade CO ^40^. However, on the night of SD, CO protein is ubiquitinated and degraded by COP1-SPA complexes. Consistently, *hos1 cop1* double mutants display complete photoperiodic insensitivity, suggesting that HOS1 and COP1 function synergistically in the control of flowering time ^41, 42^. Moreover, a regulator of the TOPOISOMERASE VI complex, MIDGET (MID), physically interacts with COP1 and is required for COP1 function as a repressor of flowering under SD conditions ^43^.

In SD plant rice, *PETER PAN SYNDROME* ( *PPS*) encodes an ortholog of *Arabidopsis COP1.* Although PPS is similar to COP1 in repressing photomorphogenesis ^44^, it controls photoperiodic flowering by HEADING DATE 1 (Hd1) (ortholog of *Arabidopsis* CO) via a currently unknown mechanism ^45^.

## Future perspectives

Extensive studies have revealed a complicated but delicate network in regulating photoperiodic flowering in plants. After the role of COP1 in repressing light responses at seedling stage by the regulation of proteolysis was established, later advances have greatly expanded its implication in the control of photoperiodic flowering and circadian rhythm. The studies mentioned in this review have also raised a number of challenging questions to be addressed in the future. As a long-term goal, the roles of COP1 in light quality control of flowering would be of great interest to determine. Specifically, how does COP1 work in concert or function antagonistically with other key factors to control CO abundance/activity in a special photoperiod or in response to multiple environmental cues? How does COP1 determine the substrates to be degraded by the COP1–SPA complex alone or together by other COP/DET/FUS protein-containing complex(es)? Moreover, the identification and characterization of novel direct targets of COP1 in the control of photoperiodic flowering will assist us in understanding the molecular mechanism underlying CO-independent pathways. In addition, further studies on the differential mechanisms of COP1 function in *Arabidopsis* and crop plants will help us to explore their functional novelty and diversity during the evolution of monocots and dicots.

## Abbreviations

AS1, ASYMMETRIC LEAVES 1; CDF, CYCLING DOF FACTOR; CO, CONSTANS; COP1, CONSTITUTIVE PHOTOMORPHOGENIC 1; CRY, cryptochromes; CUL4, CULLIN4; DET1, DE-ETIOLATE 1; FKF1, FLAVIN BINDING, KELCH REPEAT, F-BOX 1; FT, FLOWERING LOCUS T; GI, GIGANTEA; HOS1, HIGH EXPRESSION OF OSMOTICALLY RESPONSIVE GENES 1; LD, long-day; MID, MIDGET; PPS, PETER PAN SYNDROME; NF-Y, NUCLEAR FACTOR-Y; PHY *,* phytochromes; SD, short-day; SPA, SUPPRESSOR of *phyA-105*.
